# When opportunity structures matter for unfolding the sociopolitical development of adolescents and young adults

**DOI:** 10.1111/jora.70058

**Published:** 2025-08-01

**Authors:** Andres Pinedo, Nate Kruger

**Affiliations:** ^1^ Department of Human and Organizational Development Vanderbilt University Peabody College Nashville Tennessee USA

**Keywords:** critical consciousness, developmental phase, opportunity structures, sociopolitical development

## Abstract

Recognizing the significant roles of youth in social change efforts, research has increasingly aimed to clarify the processes that promote critical action among young people. Studies on sociopolitical development and critical consciousness emphasize that youth's reasoning about inequality and their sociopolitical efficacy are particularly salient precursors to critical action. However, this body of scholarship has yet to clarify the role of opportunity structures in sociopolitical development. Even less research investigates how the key factors in the sociopolitical development framework (e.g., critical reflection, sociopolitical efficacy, opportunity structures) differentially shape youth's critical action depending on their developmental phase. The current study analyzes the processes outlined in the theory of sociopolitical development using a diverse national sample of adolescents and young adults. Multigroup structural equation modeling of participants' survey data elucidated the prominent positive relationship between critical reflection and critical action, but revealed that sociopolitical efficacy was only positively linked to critical action in young adults. Additionally, the results indicated that opportunity structures played a more significant role in shaping the critical action of adolescents than that of young adults. In summary, the distinct relationships observed between adolescents and young adults highlight the necessity of considering key developmental differences in the sociopolitical development process to clarify pathways to political engagement.

## INTRODUCTION

Youth civic engagement is essential for a robust democracy, and their engagement has strengthened contemporary social movements, such as those for Native American sovereignty, environmental justice, and racial justice (Anyiwo et al., 2020; Dai et al., 2023; Flanagan, 2012). Seeing youth's tremendous roles in social change efforts, research has increasingly sought to elucidate the processes that foster youth civic engagement and broader critical action (Pinedo, Diemer, & Frisby, 2024; Wray‐Lake & Abrams, 2020). Two theoretical frameworks, sociopolitical development and critical consciousness, have been prominent in elucidating the factors that drive youth critical action (Hope et al., 2023; Pinedo, Diemer, & Frisby, 2024; Watts & Flanagan, 2007). Sociopolitical development and critical consciousness research highlight that youth's reasoning about inequality and their sociopolitical efficacy are particularly salient precursors to critical action (Diemer et al., 2021; Hope & Jagers, 2014). A robust body of research has shed light on the interrelations between critical reflection, sociopolitical efficacy, and critical action (Pinedo, Diemer, & Frisby, 2024). However, this scholarship has yet to clarify the role of opportunity structures in sociopolitical development and critical action (Heberle et al., 2020; Watts & Flanagan, 2007).

Opportunity structures refer to organized opportunities that youth have for translating their thinking about social issues into action aimed at remedying them (Watts & Flanagan, 2007). In Watts and Flanagan's (2007) model of sociopolitical development, youth's critical reasoning about inequality (i.e., critical reflection) is thought to shape their critical action, but this relationship is dependent on whether youth have opportunities to engage in action. Accordingly, youth with greater access to opportunity structures are thought to be more engaged civically than youth with fewer opportunities. Yet, little work explicitly examines the role of opportunity structures in the sociopolitical development process. Even less work examines how the key factors in the sociopolitical development framework (e.g., critical reflection, sociopolitical efficacy, opportunity structures) differentially shape youth's critical action depending on their developmental phase. Considering the differing constraints and affordances for adolescents (i.e., youth ages 13–17) compared with young adults (i.e., youth ages 18–24 years old), it is plausible that these relationships emerge differently across developmental phases (Arnett, 2000; Steinberg & Morris, 2001).

Despite important developmental differences, research on sociopolitical development often treats adolescents and young adults in the same way: as broadly “youth.” While both groups should be considered youth, this area of scholarship can be advanced by attending to experiential differences across these distinct developmental phases. In this article, youth between the ages of 13 and 17 will be termed adolescents, while those between 18 and 24 will be termed young adults, and youth and young people will be used interchangeably to describe the broader grouping of individuals between 13 and 24 years of age. The current study examines the processes presented in the theory of sociopolitical development among a diverse sample of adolescents and young adults who roughly reflect the U.S. population of youth. This sampling approach enables the current research to examine youth with diverse experiences regarding neighborhood characteristics, offering variation to understand the role of opportunity structures in the sociopolitical development of adolescents and young adults.

### Sociopolitical development: Links between critical consciousness dimensions

Central to the sociopolitical development framework is the multidimensional construct of critical consciousness, which is comprised of three dimensions: critical reflection–the awareness of systemic social inequalities and the role of historical injustices in their formation; critical action–sociopolitical action taken in opposition to inequalities; and critical motivation or sociopolitical efficacy–an individual's drive and perceived capacity to work toward social justice (Diemer et al., 2022; Watts & Flanagan, 2007). The overarching construct of critical consciousness is thought to be essential for marginalized people to reason critically about the underpinnings of inequality, develop a belief in their capacity to challenge inequality, and engage in actions that undermine social inequality (Freire, 2000; Heberle et al., 2020). For those occupying more privileged social positions, critical consciousness is thought to encourage their support for social movements that challenge social inequality (Burson & Godfrey, 2020). According to theory, there is a dynamic relationship between these dimensions of critical consciousness, with sociopolitical efficacy serving an augmenting role in channeling critical reflection into critical action (Hope et al., 2023; Suzuki et al., 2023).

Interestingly, the empirical literature reveals inconsistent findings in how these three dimensions relate to one another (Hope et al., 2020; Pinedo, Diemer, & Frisby, 2024; Pinedo, Frisby, et al., 2024). A cross‐sectional study with a nationally representative sample of youth found that sociopolitical efficacy did not mediate or moderate the association between critical reflection and various forms of critical action, failing to support theoretical postulations (Diemer & Rapa, 2016). However, two cross‐sectional studies with Black adolescents showed that critical reflection was indirectly related to critical action via critical agency (akin to sociopolitical efficacy)—lending support for the mediation hypothesis (Anyiwo et al., 2023; Hope et al., 2020). In support of the moderation hypothesis, qualitative research with young adults suggested that sociopolitical efficacy encouraged youth to act on their awareness of social issues (Guerrero et al., 2021). Furthermore, a latent profile analysis of youth's critical consciousness typologies revealed that sociopolitical efficacy was a key variable distinguishing classes of youth critical consciousness–suggestive of a moderating role (Godfrey et al., 2019).

**FIGURE 1:**
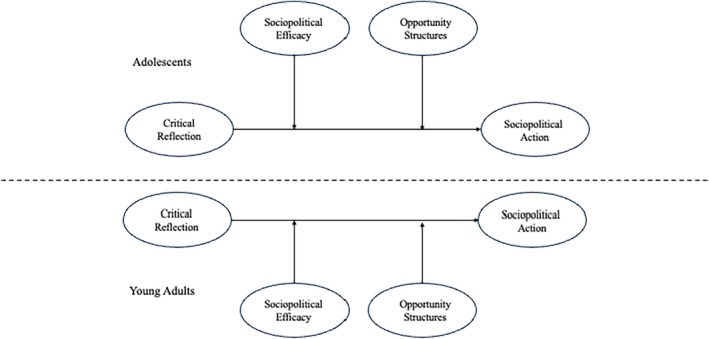
Conceptual model: sociopolitical efficacy moderates the link between critical reflection and action.

Given these empirical links between the dimensions of critical consciousness and the complexity of the sociopolitical development process, this study aims to test the model presented in Watts and Flanagan's (2007) theoretical explication. Specifically, we assess whether sociopolitical efficacy moderates the relationship between critical reflection and critical action in a diverse sample of adolescents and young adults. Importantly, we also examine whether opportunity structures, as presented in Watts and Flanagan's (2007) explication, moderate the link between reflection and action (see Figure 1).

### The role of opportunity structure in sociopolitical development

Inconsistency in how the three dimensions of critical consciousness relate to one another in past work has pushed scholars to more seriously consider the roles of opportunity structures in linking these dimensions (Heberle et al., 2020; Pinedo, Diemer, & Frisby, 2024). Given the ecological and transactional nature of sociopolitical development, it is possible that the ways in which these dimensions relate depend on the ecology of youth's lives and their interactions with this ecology (Bronfenbrenner & Morris, 2007; Watts & Flanagan, 2007). That is, the contexts in which youth develop, the opportunities for civic engagement available to them or lack thereof, and how they engage with these contexts likely affect how their sociopolitical development unfolds.

A pair of studies support this hypothesis. In one study, youth in urban areas with access to multiple opportunity structures reported increases in their critical reflection and engagement in critical action while maintaining constant and high levels of critical motivation over time (Pinedo, Frisby, et al., 2024). On the other hand, a separate study that sampled youth in rural communities enrolled in ethnic studies found that these youth, with few or no opportunity structures (e.g., youth activist organizations), reported no change in their engagement in critical action and declining critical motivation, despite showing growing critical reflection (Pinedo et al., 2025). Importantly, the broader literature on civic engagement highlights that differences in youth civic engagement can be traced to the presence of opportunity structures, which are rarely provided to marginalized communities (Carnegie Corporation of New York, 1992; Keeter et al., 2002; Smith, 1999).

When youth do have access to these opportunity structures for political action, they report greater sociopolitical development, and importantly, critical reflection and sociopolitical efficacy positively relate to critical action (Hipolito‐Delgado et al., 2024). For example, past research reveals that individuals with access to active community organizing groups engage more in critical action than those with less access (Christens & Speer, 2011). In a nationally representative longitudinal study, youth's opportunities for civic engagement and actual engagement in high school predicted civic engagement at an 8‐year follow‐up (Hart et al., 2007). Notably, it appears that early opportunities for critical action set youth up to remain politically engaged in the transition to adulthood (Flanagan et al., 2009; Flanagan & Levine, 2010).

### Attending to developmental phases in the unfolding of sociopolitical development

Opportunities for political engagement may be particularly salient for adolescents compared with young adults (Flanagan et al., 2009). The period of adolescence is characterized by a particular sensitivity to external influences from peers, family, and community (Bronfenbrenner & Morris, 2007; Wray‐Lake, 2019). Adolescents are especially influenced by their peers, which may make spaces in which they can act collectively with their peers especially catalytic for their critical action (Tyler et al., 2020). According to the developmental cascades model, the effects of complex systems on youth's development vary across development, and these effects have cascading effects such that earlier experiences and learned capacities accumulate over time to shape later outcomes (Chaku & Davis‐Kean, 2024; Cicchetti & Rogosch, 2009). As such, it is likely that opportunity structures for critical action are especially salient for adolescents who are particularly sensitive to community influences (Ginwright & Cammarota, 2002; Wray‐Lake, 2019). If provided opportunities for political engagement, youth can establish a habit of channeling reflection on inequality into critical action, which can positively shape their sociopolitical development over the transition into adulthood (Flanagan et al., 2009).

Given adolescents are at a developmental peak for habit formation, opportunity structures for critical action may lay the groundwork for long‐term engagement in this realm (Flanagan & Levine, 2010; Hart et al., 2007). In developmental cascades parlance, early exposure to opportunity structures can have cascading effects and increase their likelihood of sustained participation in critical action (Chaku & Davis‐Kean, 2024; Masten & Cicchetti, 2010). For example, adolescents who engage in community organizing may develop greater sociopolitical efficacy, reinforcing their likelihood of continued engagement in adulthood (Kirshner & Ginwright, 2012; Pinedo, Frisby, et al., 2024). These early experiences can set off a cascade in which critical reflection, sociopolitical efficacy, and networks accumulate over time, making sustained action more likely. Thus, from a developmental cascades perspective, sociopolitical development is shaped by the interplay between early experiences, accumulating capacities, and the structural conditions that either facilitate or constrain engagement over time. Given adolescents' developmental propensities, constraints on their ability to move freely without parental consent, and barriers to political engagement (i.e., voting age requirements), opportunity structures are likely to be a more potent predictor of their critical action than those for young adults.

Young adults gain greater independence as they become legal adults at 18 and have access to the traditional political sphere (Flanagan & Levine, 2010; Watts & Flanagan, 2007). Researchers argue that civic engagement organically develops amidst the transition from adolescence to emerging adulthood, as individuals develop the community ties and social roles necessary for civic engagement (Flanagan & Levine, 2010). Longitudinal research from Western countries found political engagement to steadily increase throughout the transition to early adulthood (Claes & Hooghe, 2017; Wray‐Lake et al., 2020). With increased independence and the ability to move around freely, young adults' engagement in critical action may be less bound by the immediate availability of opportunity structures in their communities than that of adolescents'.

Despite increasing freedom to engage politically, young adulthood may present barriers to critical action. For instance, young adults, particularly those from marginalized backgrounds, often need to work more to sustain themselves, which places limits on their time (Arnett, 2000; Guerrero et al., 2021). Considering specific barriers and potential developmental cascades, young adults' critical action may be particularly tied to their sociopolitical efficacy (Leath & Chavous, 2017). Broadly, young adults who successfully engage in critical action during adolescence may be more likely to act during emerging adulthood because their past experiences communicate that they can engage in impactful change‐making.

### The current study

Altogether, this study empirically tests the model presented in the theory of sociopolitical development (Watts & Flanagan, 2007). Specifically, we assess whether youth's perceptions of the opportunity structures available to them within their community support translating critical reflection into critical action. Crucially, however, and drawing on a developmental phase perspective, we examined whether opportunity structures are more important for catalyzing critical action among adolescents than young adults. We suspect this developmental difference given the unique nature of adolescence, in which adolescents are especially influenced by their peers but face more barriers to their political engagement. Meanwhile, young adults gain more autonomy due to their adult legal status and increased mobility. Further, we also assess whether sociopolitical efficacy strengthens the relationship between critical reflection and critical action. In sum, we statistically test the complete theoretical model of sociopolitical development, inclusive of opportunity structures in a national sample for the first time. By presenting a detailed picture of relationships between sociopolitical development dimensions among two developmental cohorts, we lend essential theoretical clarity to a burgeoning field of research. Overall, we sampled diverse youth from across the U.S. to assess the following research questions and related hypotheses:

Q1: What are the associations between critical reflection and sociopolitical efficacy with critical action among adolescents and young adults?
  H1: Adolescents and young adults' critical reflection will be positively associated with their engagement in critical action.
  H2: Sociopolitical efficacy will be more strongly associated with young adults' critical action than with adolescents' critical action.
Q2: Does sociopolitical efficacy moderate the relationship between critical reflection and critical action for both adolescents and young adults?
  H3: Sociopolitical efficacy will moderate the relationship between critical reflection and critical action for both adolescents and young adults.
Q3: How are participants' perceptions of opportunity structures related to their critical action, and do perceptions of opportunity structures moderate the relationship between critical reflection and critical action among adolescents and young adults?
  H4: Both adolescents' and young adults' perceptions of opportunity structures will be positively related to their engagement in critical action.
  H5: Perceptions of opportunity structures will moderate the relationship between critical reflection and critical action among adolescents but not young adults.

## METHOD

Data for this study are drawn from a 2024 election study designed to represent adolescents and young adults across the United States (U.S.). Data collection occurred over the 2 weeks preceding the U.S. presidential election of 2024. Overall, the sample comprises 427 participants, with 274 young adults and 155 adolescents. Participants represented 44 of the 50 U.S. states. Participants' mean age was 18.9. 28.7% of the sample was Black, 17.9% was Latinx, 34.1% was White, 7.9% was Asian, 7.2% was Multiracial, 0.7% was Middle Eastern, and 3.3% was Native American. Table 1 displays participant demographics broken down by age group.

**TABLE 1:** Demographic statistics (% in Group).

	Adolescents (*M*_age_ = 14.6)	Emerging adults (*M*_age_ = 21.4)
**Gender**		
Woman	49.7	50
Man	50.3	49.3
Non‐binary	0	0.7
**Race**		
Black	25.2	30.8
Latinx	17.4	18.1
White	39.4	31.1
Asian	7.1	8.4
Native American	3.2	3.3
Middle Eastern	0	1
Multiracial	7.7	7
**Household income**		
≤$30,000	23.9	22.3
$30,001–$70,000	22.6	33.9
$70,001–$110,000	18.7	11.7
$110,001–$170,000	11.0	14.2
$170,000+	15.5	12.4
Don't know	8.4	5.5

### Procedure

Qualtrics Panels, a participant recruitment and survey company, was contracted to recruit participants from all over the United States via online recruitment. Specifically, they sent study invitations via their recruitment app “Surveys on the GO” to participants in their pool who fit the study's recruitment criteria. These criteria stipulated that potential participants needed to be between 13 and 24 years old and reside in the United States. Though young adult participants (i.e., age ≥ 18) were contacted directly, adolescent participants (i.e., age < 18) were contacted through their parents. That is, the adolescents' parents received the invitation and associated consent form, and upon parental consent, adolescent participants were sent a survey link that included an assent form. Young adult participants' invitation included the consent form, and they provided consent prior to obtaining the survey link. The recruitment period for Wave 1 data collection spanned the 2 weeks preceding the 2024 U.S. Presidential Election. The survey took ~ 10 min to complete. All participants received an incentive, which varied depending on their individual agreement with Qualtrics Panels. All of this study's procedures were approved by the Institutional Review Board at the author's home institution.

### Measures

#### Critical action

We assessed critical action using the critical action subscale of the Short Critical Consciousness Scale (ShoCCS; Diemer et al., 2022). This subscale consists of five items that asked participants to indicate their retrospective engagement in critical action. Specifically, they were instructed to indicate how often they engaged in a set of critical actions over the past year. A sample item is, “Joined in a protest march, political demonstration, or political meeting.” Participants responded to these items on a 1 (never did this) to 5 (at least once a week) Likert scale. The Cronbach's alpha for this scale was 0.95. Table 2 includes descriptive statistics for each of the items and restates the Cronbach's alpha.

**TABLE 2:** Descriptive statistics for adolescents and emerging adults.

Latent variable/indicators	Adolescents			Emerging adults		
	*M*	SD	Skew	*M*	SD	Skew
**Critical reflection (*α*A = 0.87; *α*EA = 0.87)**						
1: Certain racial or ethnic groups have fewer chances to get good jobs	4.26	1.25	−0.45	4.31	1.33	−0.67
2: Certain racial or ethnic groups have fewer chances to get ahead	4.13	1.31	−0.37	4.26	1.32	−0.69
3: Women have fewer chances to get ahead	3.95	1.35	−0.27	4.06	1.42	−0.51
4: Poor people have fewer chances to get ahead	4.43	1.32	−0.59	4.42	1.44	−0.82
**Political Efficacy (α**_**A**_ **= 0.82; α**_**EA**_ **= 0.85)**						
1: Create a plan to address the problem	3.70	0.94	−0.12	3.68	1.05	−0.43
2: Organize and run a meeting	3.39	1.15	−0.20	3.38	1.14	−0.14
3: Write an opinion letter to a local newspaper	3.78	1.06	−0.62	3.71	1.09	−0.54
**Critical Action (α**_**A**_ **= 0.93; α**_**EA**_ **= 0.95)**						
1: Participated in a civil rights group or organization	2.00	1.25	0.81	2.12	1.36	0.77
2: Participated in a political party, club, or organization	2.03	1.31	0.83	2.11	1.35	0.81
3: Contacted a public official by phone, mail, or email to tell him or her how you felt about a social or political issue	2.05	1.29	0.87	2.01	1.29	1.00
4: Joined in a protest march, political demonstration, or political meeting	2.14	1.33	0.70	2.05	1.35	0.97
5: Participated in a human rights, gay rights, or women's rights organization or group	1.97	1.29	1.06	2.03	1.25	0.83
**Opportunity Structures (α**_**A**_ **= 0.83; α**_**EA**_ **= 0.87)**						
1: In your view, how many political and or civic organizations exist in your community?	2.57	0.84	−0.25	2.52	0.91	−0.05
2: In your view, how many events are there in your community that encourage civic participation, such as town hall meetings or public forums?	2.47	0.88	−0.26	2.52	0.95	−0.14
3: In your view, how many people are organizing opportunities for political involvement in your community?	2.39	0.90	−0.14	2.51	0.95	−0.11

#### Sociopolitical efficacy

We assessed sociopolitical efficacy using an adapted measure from Flanagan et al. (2009) Civic Measurement Models working paper for CIRCLE. Our adapted measure used three items, which asked participants how well they could do a series of civic tasks that could help them address a community issue. A sample item is “organize and run a meeting.” Participants answered these items on a 1 (definitely can't) to 5 (I definitely can) Likert scale. Cronbach's alpha for this scale was 0.82. Table 2 includes descriptive statistics for each item.

#### Critical reflection

In this study, we measured critical reflection with the critical reflection subscale of the ShoCCS (Diemer et al., 2022). This subscale consists of four items that gauge participants' attributions (i.e., individual versus structural) for inequality. Participants were instructed to report how much they agreed or disagreed with each of the items. Sample items include “Certain racial or ethnic groups have fewer chances to get good jobs” and “Women have fewer chances to get ahead.” Participants indicated their agreement on a 1 (strongly disagree) to 6 (strongly agree) Likert scale. Cronbach's alpha for this scale was 0.87. As with all measures, Table 2 includes descriptive statistics for each item.

#### Opportunity structures

We measured opportunity structures using a set of newly developed items guided by existing theory and research. The three Likert items assessed participants' perceptions of opportunities for political and civic participation in their neighborhoods. Sample items include “In your view, how many events are there in your community that encourage civic participation, such as town hall meetings or public forums?” and “In your view, how many people are organizing opportunities for political involvement in your community?” Participants responded on a four‐point scale ranging from 1 (less than I'd like) to 4 (more than enough). Cronbach's alpha for this scale was 0.83. Table 2 includes descriptive statistics for each item.

### Analytic plan

The analytic plan for this study followed the typical two‐step approach to structural equation modeling (Kline, 2023a, 2023b). Structural equation models were fit using RStudio and Lavaan (Rosseel, 2012). Specifically, we first fit a measurement model via confirmatory factor analysis (CFA) to assess how well the survey items (i.e., indicators) loaded onto their specified latent constructs (Bollen & Hoyle, 2012; Kline, 2023a, 2023b). CFA parses measurement error from true score error, enabling the assessment of the relationships of interest while attenuating the effects of measurement error (Bollen, 2002). After ascertaining a well‐fitting measurement model, we estimated the relationships between the three dimensions of critical consciousness and opportunity structures using their latent variable forms–moving into a full structural equation model. Maximum likelihood was used as the estimation method, given that all the study variables were treated as continuous. Full information maximum likelihood was employed to handle missing data, enabling the use of all available data rather than deleting incomplete data (Enders, 2023). Model fit, a key advantage of structural equation modeling, was determined via the comparative fit index (CFI), Tucker–Lewis index (TLI), root mean square error of approximation (RMSEA), and standardized root mean residual (SRMR). These indices convey the degree to which the proposed model reflects the underlying data (Kline, 2023a, 2023b). We followed standard interpretive practice for assessing fit in which a CFI ≥ 0.95, TLI ≥ 0.95, RMSEA ≤ 0.06, and SRMR ≤ 0.08 reflect good fit to the data (Hu & Bentler, 1999). Nonetheless, contemporary perspectives argue there are no universal cut‐off values for good fit and that all these indices, together with model parameters, should be judged collectively to determine the adequacy of the model (West et al., 2012).

A central goal of the current study was to test a moderation hypothesis, in which the relationship between critical reflection and critical action depends on the availability of opportunity structures within the participants' contexts. Moderation, or interaction effects, have historically been difficult to estimate with latent variables in the structural equation modeling framework (Maslowsky et al., 2015). However, we implemented these moderation analyses using the “modsem” package in Rstudio, recently developed to aid in the estimation of latent variable interactions (Slupphaug et al., 2024).

Using modsem, we estimated our hypothesized interaction effects with the double mean centering approach (Lin et al., 2010; Slupphaug et al., 2024). In the double mean centering approach, the indicators are centered prior to estimating the product terms, which eliminates collinearity and thus results in more accurate interaction and main effects (Slupphaug et al., 2024). The modsem package also leverages Monte Carlo simulation to produce a model‐implied covariance matrix, which has historically been challenging to estimate for latent variable interaction models (Schoeman & Jorgenson, 2021; Slupphaug et al., 2024). In doing so, this package produces fit indices for these nonlinear interaction models, which have been absent in previous approaches to estimating interaction models, presenting another advantage of leveraging modsem for the current analyses.

Finally, the primary aim of the current study was to determine whether opportunity structures have varying significance depending on youth's phase of development, particularly whether opportunity structures are more important for adolescents as compared with young adults. We implemented a multigroup model to assess this hypothesis. Multigroup structural equation modeling permits the estimation of a model for two groups separately to test whether the hypothesized relationships vary across groups (Kline, 2023a). To ensure group differences reflected true differences in the nature of relationships, rather than artifacts of unequal measurement, we first assessed measurement invariance (Van de Schoot et al., 2012). We followed the traditional approach to measurement invariance testing, where we first fit the configural model with a CFA that included all four central constructs, then the metric invariance model, followed by the scalar invariance model, and finally the residual invariance model (Kline, 2023a). After ascertaining measurement invariance, we estimated the multigroup model to test the primary hypothesis.

While our overarching analytic approach is well‐suited for the study's aims, we acknowledge that our sample size and, thereby, statistical power are a potential limitation of the current analysis. Latent variable interaction models typically require larger samples due to the complexity of estimating nonlinear effects. As such, these results must be interpreted with consideration for the smaller‐than‐ideal sample size. Nonetheless, the modsem package estimates these models using Monte Carlo estimation, which improves estimate precision and stability in smaller samples (Slupphaug et al., 2024). Furthermore, we estimated these models with strong latent factor models, which attenuate the consequences of measurement error and increase statistical power and estimate precision (Kline, 2023a).

## RESULTS

### Descriptive statistics

Prior to estimating the study's primary models, we assessed descriptive statistics (e.g., means, standard deviations) for the entire sample, as well as separately for adolescents and young adults. These descriptive results are reported in Table 1. Importantly, our measurement invariance analyses revealed that our measures were invariant across adolescents and young adults, as indicated by unchanging fit indices across invariance models, suggesting that these groups interpreted the measures in the same manner. Thus, comparisons can be made between structural relationships (see Table 3).

**TABLE 3:** Fit statistics for measurement models.

Model	*Χ* ^2^	df	*Χ* ^2^Δ	RMSEA	CFI	TLI	SRMR
Configural Invariance	257.14	168	NA	0.04	0.98	0.98	0.04
Metric Invariance	264.23	179	7.09	0.04	0.98	0.98	0.04
Scalar Invariance	277.93	190	13.70	0.04	0.98	0.98	0.05

Abbreviations: CFI, comparative fit index; df, degrees of freedom; RMSEA, root mean square error of approximation; SRMR, standardized root mean square residual; TLI, Tucker–Lewis Index.

### Primary models

The model assessing hypothesis 1 and hypothesis 2 was an excellent fit to the data: (χ^2^ = 229.87 (168), *p* = .001, CFI = 0.98; TLI = 0.98; RMSEA = 0.043 CI [0.027, 0.057]; SRMR = 0.04). Adolescents' degree of critical reflection was positively related to their engagement in critical action (*β* = 0.19, SE = 0.08, *p* = .02). However, adolescents' degree of sociopolitical efficacy was not related to their engagement in critical action (*β* = 0.08, SE = 0.13, *p* = .38). For young adults, both their degree of critical reflection (*β* = 0.25, SE= 0.06, *p* < .001) and their degree of sociopolitical efficacy were associated with more engagement in critical action (*β* = 0.25, SE = 0.09, *p* < .001).

**FIGURE 2:**
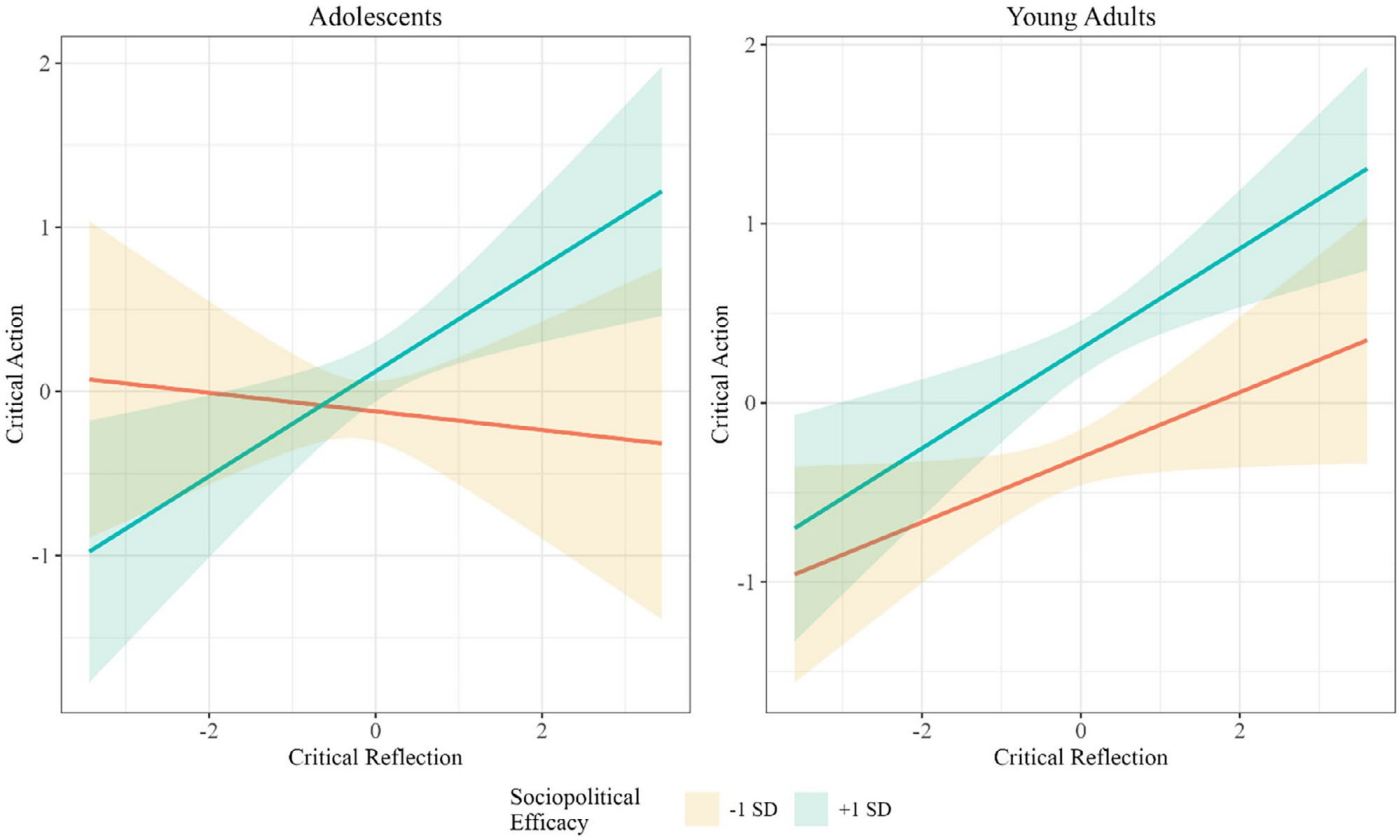
Critical reflection predicting critical action moderated by sociopolitical efficacy among adolescents and young adults.

Regarding hypothesis 3, this model was a good fit to the data (χ^2^ = 951.63 (568), *p* < .001, CFI = 0.95; TLI = 0.96; RMSEA = 0.057 CI [0.051, 0.063]; SRMR = 0.05). Interestingly, among adolescents, their sociopolitical efficacy appeared to moderate the relationship between critical reflection and critical action (*β* = 0.16, SE = 0.14, *p* = .08), but as reported, this moderation effect only approached statistical significance (see Figure 2). On the other hand, among young adults, sociopolitical efficacy seemed to play no role in shaping the relationship between critical reflection and critical action (*β* = 0.05, SE = 0.07, *p* = .43).

**FIGURE 3:**
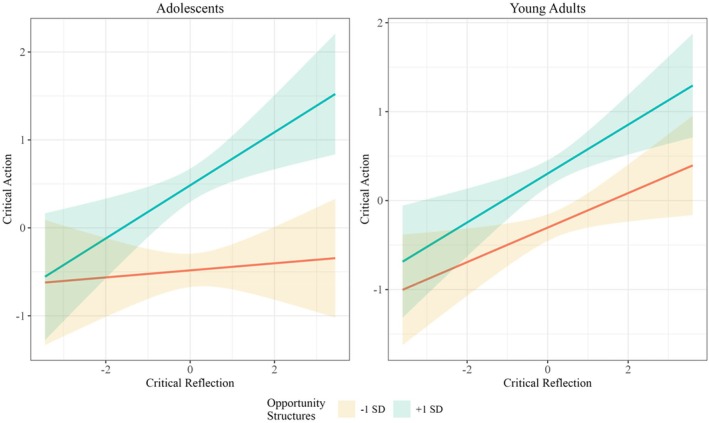
Critical reflection predicting critical action moderated by perceptions of opportunity structures among adolescents and young adults.

Lastly, the model assessing hypotheses 4 and 5 fit the data well (χ^2^ = 1170.87 (620), *p* < .001, CFI = 0.94; TLI = 0.93; RMSEA = 0.065 CI [0.059, 0.071]; SRMR = 0.05). Opportunity structures mattered for both adolescents (*β* = 0.50, SE = 0.13, *p* < .001) and young adults (*β* = 0.28, SE = 0.10, *p* < .001). However, a Wald test revealed that there was a significant structural difference between these two groups, in which opportunity structures were a stronger predictor of adolescent critical action than for young adults (*W* = 5.51 (1), *p* = .02). Furthermore, for adolescents, greater perceptions of opportunity structures strengthened the relationship between critical reflection and critical action (*β* = 0.16, SE = 0.11, *p* = .04). Among young adults, their perceptions of opportunity structures did not affect their relationship between critical reflection and critical action (*β* = 0.01, SE = 0.08, *p* = .82). A simple slopes analysis indicated that for adolescents who perceived few opportunity structures in their community (i.e., −1 SD), every one‐unit increase in critical reflection was associated with a 0.04 increase in critical action (*β* = 0.05, *p* < .001). In contrast, for adolescents who perceived many opportunity structures in their community (i.e., +1 SD), every one‐unit increase in critical reflection was associated with a 0.35 increase in critical action (*β* = 0.35, *p* < .001). This moderation analysis supported hypothesis 5, highlighting that opportunity structures play a more significant role for adolescents than young adults (see Figure 3).

## DISCUSSION

This study advances the literature on critical consciousness and sociopolitical development by testing whether sociopolitical efficacy and opportunity structures moderate the relationship between critical reflection and action, and whether these structural relationships differ between adolescents and young adults. Incorporating a developmental cascades perspective into the literature on sociopolitical development, we hypothesized that opportunity structures would play a more significant role in the critical action of adolescents, while sociopolitical efficacy would have a greater impact on young adults' critical action (Chaku & Davis‐Kean, 2024; Flanagan, 2012). These hypotheses were based on research into fundamental developmental processes, which emphasize that adolescents are especially responsive to contextual influences (Flanagan & Levine, 2010; Ginwright & Cammarota, 2002). Among young adults, earlier experiences with critical action may positively influence sociopolitical efficacy, making it a more powerful catalyst for their critical action (Flanagan, 2012; Youniss et al., 2002).

Lending support for our first hypothesis, our findings revealed that adolescents and young adults with higher levels of critical reflection were more engaged in critical action. This aligns with a fundamental principle of the sociopolitical development framework, which views a critical analysis of inequality as a crucial precursor to critical action (Hipolito‐Delgado et al., 2024; Hope et al., 2020; Watts & Flanagan, 2007). Interestingly, and consistent with hypothesis 2, sociopolitical efficacy was only positively associated with critical action among young adults. It is possible that sociopolitical efficacy influences young adults' critical action because their past experiences inform their sociopolitical efficacy and thus give it greater significance, while adolescents' sociopolitical efficacy has yet to be shaped by previous experiences. This would align with the developmental cascades perspective, that early experiences and learned capacities accumulate over time to shape later outcomes (Cicchetti & Rogosch, 2009; Zaff et al., 2008).

Of note, while sociopolitical efficacy was a significant predictor of young adults' critical action, young adults' sociopolitical efficacy did not affect (i.e., moderate) the association between their critical reflection and action. This suggests that critical reflection and sociopolitical efficacy are independent predictors of critical action and that their effects on action are not dependent on each other (Hipolito‐Delgado et al., 2024; Hope & Jagers, 2014). For adolescents, however, while their sociopolitical efficacy did not predict their critical action independently, it seemed to moderate the relationship between their critical reflection and critical action, as this interaction effect approached statistical significance. These distinct sets of relationships among adolescents and young adults suggest that attending to key developmental differences in the sociopolitical development process can clarify inconsistent associations across studies (Diemer & Rapa, 2016; Heberle et al., 2020; Leath & Chavous, 2017).

Significantly, our findings indicated that perceptions of opportunity structures were positively linked to engagement in critical action for both groups, with a stronger association for adolescents' critical action compared with young adults. A significant moderation effect underscored the importance of opportunity structures for adolescents (Diaz et al., 2023). Specifically, opportunity structures strengthened the relationship between critical reflection and critical action, but only for adolescents—thereby supporting hypothesis 4. The significance of opportunity structures for adolescents aligns with the developmental literature, which emphasizes that adolescence is a period in which political awareness and involvement sprout and contextual influence is particularly strong (Flanagan, 2012; Hart et al., 2007; Wray‐Lake, 2019). This further underscores the utility of the developmental cascades model for the sociopolitical development process, as it reflects the idea that systems (e.g., opportunity structures) have varying impacts across development (Chaku & Davis‐Kean, 2024; Cicchetti & Rogosch, 2009).

### Implications

Together, these findings offer important lessons for practice and theory regarding youth sociopolitical development and critical consciousness. Recently, the field has increasingly investigated the role of consciousness‐raising systems in the sociopolitical development process (Heberle et al., 2020; Pinedo, Frisby, et al., 2024). Consciousness‐raising systems refer to spaces that encourage a structural analysis of inequality and offer scaffolded opportunities for critical action, but often are more focused on fostering reflection than action (Pinedo et al., 2025; Watts & Hipolito‐Delgado, 2015). Our findings suggest that if organizations aim to facilitate students' engagement in critical action, then they must provide relevant opportunities for critical action (Zaff et al., 2008). Furthermore, given the increasing importance of sociopolitical efficacy for action across development, it may be beneficial for organizations to provide adolescents with opportunities for action that are more likely to have an impact, allowing them to build their sociopolitical efficacy in the early stages of their involvement.

Moreover, this study offers important insights for theoretical innovation. Specifically, the significantly different structural relationships across adolescents and young adults highlight that research on sociopolitical development can be advanced by teasing apart developmental differences, even among cohorts that are often grouped together (e.g., adolescents and young adults). Watts and Flanagan (2007) defined youth as individuals between the ages of 15 and 25. However, these findings suggest that this grouping may be overly broad and thus overlook important developmental differences that make some factors more important than others for sociopolitical development across these developmental periods.

### Limitations and future directions

The current study advances the literature on sociopolitical development and critical consciousness in important ways yet is hindered by some limitations. First, it draws inferences that speculate about causal processes (e.g., the nature of the association between critical reflection and critical action) using cross‐sectional observational data. Future work aiming to elucidate the directionality of these relationships and better test the sociopolitical development process can leverage longitudinal methods as previous studies have done to test these directional hypotheses (Pinedo, Frisby, et al., 2024; Suzuki et al., 2023).

Second, this study used perceptions of opportunity structures to illuminate their importance for sociopolitical development. While perceptions are indeed consequential for behavior across a host of domains (McAdam et al., 1996), future research should attempt to measure the actual availability of opportunity structures within participants' communities. A promising method may be a previously used data science approach that harnessed tax records from nonprofit civic organizations to map the availability of civic opportunity in the United States (de Vries et al., 2024).

Third, this study assessed potential differences in sociopolitical development processes according to individuals' developmental phase. In finding that these developmental processes differ across the lifespan, we suggest future research should explicate other potential developmental phase differences in sociopolitical development. For example, we found that sociopolitical efficacy appears to gain importance as an independent predictor of critical action as individuals age. Our findings suggest that a lifespan approach to studying critical consciousness and sociopolitical development is essential to draw a more comprehensive picture of these developmental processes (Baltes et al., 1999).

## CONCLUSION

Developmental research has increasingly aimed to uncover the processes that shape young people's political engagement, emphasizing their essential roles in movements for democracy and social justice (Pinedo, Diemer, & Frisby, 2024; Wray‐Lake, 2019). The current study builds on this scholarship and contributes to the extensive literature on sociopolitical development by replicating the well‐established positive relationship between critical reflection and critical action (Diemer & Rapa, 2016; Hipolito‐Delgado et al., 2024; Hope et al., 2020). Notably, the findings revealed significant developmental differences, showing that sociopolitical efficacy played a crucial role in shaping critical action among young adults, but not adolescents. Conversely, opportunity structures were more influential in translating adolescents' critical reflection into critical action. Considered together, these results highlight the importance of accounting for developmental phases in the sociopolitical development process. Young people have historically been at the forefront of social justice movements, and this study demonstrates that, given the right opportunities for political engagement, they will continue to play a major role in our future.

## CONFLICT OF INTEREST STATEMENT

The authors confirm no conflicts of interest.

## CONSENT

Participant consent was obtained from those over the age of 18, for participants under 18, parental consent was obtained along with participant assent.

## Data Availability

Research data are not shared.
